# Local excision and radical excision for rectal gastrointestinal stromal tumors: a meta-analysis protocol

**DOI:** 10.3389/fonc.2023.1224725

**Published:** 2023-09-07

**Authors:** Wenjun Luo, Chunyu Liu, Luyin Han, Haidong Zhang, Chaoyong Shen, Xiaonan Yin, Zhou Zhao, Mingchun Mu, Tianxiang Jiang, Zhaolun Cai, Bo Zhang

**Affiliations:** ^1^ Department of General Surgery, West China Hospital, Sichuan University, Chengdu, China; ^2^ Gastric Cancer Center, West China Hospital, Sichuan University, Chengdu, Sichuan, China; ^3^ Department of Pharmacy, Evidence-based Pharmacy Center, West China Second University Hospital, Sichuan University, Chengdu, China; ^4^ Intensive Care Unit, West China Hospital, Sichuan University, Chengdu, Sichuan, China; ^5^ Department of Gastrointestinal Cancer Center, Chongqing University Cancer Hospital, Chongqing, China

**Keywords:** gastrointestinal stromal tumors, GIST, rectal, local excision, radical excision

## Abstract

**Background:**

To date, several studies have compared the surgical and oncological outcomes of local excision (LE) and radical excision (RE) for rectal gastrointestinal stromal tumors (GISTs), but some have limited numbers of small series. This protocol outlines the planned scope and methods for a systematic review and meta-analysis that will compare the surgical and oncological outcomes of LE and RE in patients with rectal GISTs.

**Methods:**

This protocol is presented in accordance with the PRISMA-P guideline. PubMed, Embase, Web of Science, Cochrane Library and Wanfang database will be systematically searched. Furthermore, reference lists of all included articles will be screened manually to add other eligible studies. We will include randomized controlled trials (RCTs) and non-randomized studies (NRS) in this study. The primary outcomes evaluated will be R0 resection rate and disease-free survival, while the secondary outcomes will contain overall survival, length of stay, tumor rupture rate and complications. Two reviewers will independently screen and select studies, extract data from the included studies, and assess the risk of bias of the included studies. Preplanned subgroup analyses and sensitivity analyses are detailed within this protocol. The strength of the body of evidence will be assessed using GRADE

**Discussion:**

This review and meta-analysis will provide a comprehensive evaluation of the current evidence concerning the application of LE and RE in patients with rectal GISTs. The findings from this review will serve as a foundation for future research and emphasize the implications for clinical practice.

**Systematic review registration:**

PROSPERO (CRD42017078338), https://www.crd.york.ac.uk/PROSPERO/display_record.php?RecordID=387409, PROSPERO CRD42017078338.

## Introduction

Gastrointestinal stromal tumors (GISTs) arise from the Cajal stromal cells of the gastrointestinal tract (GI) and are the most common type of mesenchymal stromal tumors in the digestive tract (1). GISTs can occur anywhere from the esophagus to the anus but are predominantly found in the stomach (60-70%), followed by the small bowel (20-30%), and approximately 5% in the rectum (2). Although rectal GISTs are less frequent, their malignancy risk is higher than at other sites, making the rectum a worse prognostic factor for GISTs (3).

Surgical resection is the only curative treatment for primary localized GISTs (4, 5). As GISTs seldom have lymph node metastasis, complete resection with negative margins is sufficient, and routine lymphadenectomy is not necessary (6). However, the optimal surgical modality for rectal GISTs remains controversial due to the critical anatomical area, anus preservation, and postoperative urogenital nerve function (7).

Two types of surgical methods are used for rectal GIST resection: local excision (LE) and radical excision (RE). LE has the advantages of less trauma and quicker recovery but poses a higher risk of incomplete resection and disrupting the tumor capsule, potentially increasing the probability of local recurrence (8). RE can achieve complete resection with clear margins but often results in significant early and/or long-term morbidity and a worse quality of life (9). The standard treatment for rectal GISTs has not been developed, and the choice between local and radical excision for localized rectal GISTs is largely influenced by clinician preference and experience (10).

To date, several studies have compared the surgical and oncological outcomes of LE and RE for rectal GISTs (11–14), but some have limited numbers of small series. This study aims to systematically review available literature to compare the impact of LE and RE in localized rectal GIST patients on R0 resection, operation duration, length of stay, recurrence, and survival.

## Methods

### Protocol and registration

This present protocol has been registered at PROSPERO (ID: CRD42017078338). The protocol follows the reporting guidance provided in the Preferred Reporting Items for Systematic Review and Meta-Analysis Protocols (PRISMA-P) statement (see PRISMA-P checklist in Additional file 1 (15)) and will be conducted according to the PRISMA 2020 statement and the standard methodology recommended by the Cochrane Collaboration (16–18).

### Inclusion criteria

The inclusion criteria follow the PICOS (population, intervention, comparators, outcomes, and study design) framework (19). All studies meeting the inclusion criteria will be included without restrictions on language and publication year.

**• Population:** Patients with primary rectal GISTs who underwent surgical resection and had GISTs confirmed by pathological results (Immunohistochemical assay for SDHB, CD117, DOG1, and CD34 and molecular genetic testing for KIT and PDGFRA mutations, as well as other potential drivers (e.g., BRAF, NF1, NTRK, and FGFR fusions) will be included without restrictions on country, race, ethnic origin, age, sex, or occupation (20).

**• Intervention:** Local excision includes transanal excision, transanal minimally invasive surgery, transanal endoscopic microsurgery, trans-sacral resection, and transperineal resection.

**• Comparators:** Radical excision includes abdominal-perineal resection, low anterior resection, and Hartmann operation.

**• Outcomes and measurement:**

**Primary outcome:** 1.R0 resection rate; 2. disease-free survival.

**Secondary outcome:** 1.overall survival; 2. length of stay; 3. tumor rupture rate; 4. complications.

**• Study designs:** The study will also include non-randomized studies (NRS) (case–control studies, and cohort studies) and randomized controlled trials (RCTs). The included studies must report at least one of the prescriptive outcomes in rectal GIST patients who undergo any form of surgery.

### Exclusion criteria

– The presence of metastatic, recurrent or other secondary rectal GISTs.
– Duplicate texts and articles.
– Articles with incomplete clinical data after a reasonable attempt at contacting corresponding authors.
– Any letters, conference abstracts, editorials, case reports, reviews or nonclinical studies without available data will be excluded.
– Full-text articles cannot be obtained after exhaustive searches.

### Information sources and search strategy

A systematic literature search will be performed in PubMed, Embase, Cochrane Central Registry of Controlled Trials to identify all relevant studies from their inception to Sep 10th, 2023. The details of PubMed database search strategy are presented in Additional File 2. Reference lists of all included articles will be screened manually to add additional studies if they meet the eligibility criteria and full text will be retrieved.

### Study selection and data extraction

All study records obtained by literature search will be imported in EndNote software. After removing duplicates, two reviewers (L.W and L.C) will conduct articles selection independently based on the eligibility criteria outlined above. First of all, titles and abstracts will be screened for relevance. Next, the two independent reviewers (L.W and L.C) will reassess all potentially relevant full-text articles. The articles screening process will be summarized in a PRISMA flow diagram (21).

Two reviewers (L.W and L.C) will independently complete data extraction for the included studies using a standardized data extraction form. The collected data will include baseline, disease and perioperative characteristics of patients, as well as data relevant to our primary and secondary outcomes. A third reviewer (Z.B) will make the final decision if any conflicts occur in the process of study selection and data extraction which couldn’t be resolved by the two reviewers.

### Dealing with missing data

When some required data is missing, we will contact the corresponding author of the article via email. If no response is received in 14 days after the initial contact, we will no longer communicate with the study researcher, and the selected studies will be excluded from the present systematic review and meta-analysis.

### Risk of bias assessment

Two independent reviewers (L.W and L.C) will also assess methodological quality/risk of bias of the included studies at the individual study level, and disagreements will be resolved through discussion or by a third reviewer (Z.B) (22). NRS will be assessed using Risk of Bias in Non-randomized Studies of Interventions (ROBINS-I) tool (23), while RCTs will be assessed risk of bias with the RoB 2 (24).

### Data synthesis

Two reviewers (L.W and L.C) will independently extract data from the included studies, including publication year, primary author name, journal of publication, study type, number of patients, tumor size, type of surgery, surgical approach, margin status, length of stay, and survival. Additional data, such as tumor rupture rate, will also be recorded. Conflicts will be resolved through discussion or by involving a third reviewer (Z.B). If sufficient data is available for a quantitative synthesis, a meta-analysis will be conducted using STATA version 15.0 statistical software (STATA, College Station, TX). Binary data will be reported as odds ratios (OR) with 95% confidence intervals (95% CI) estimated using the Mantel-Haenszel method. For continuous data, mean differences and 95% CI will be estimated using inverse variance weighting. Outcome measures (mean + standard deviation and median + interquartile range) will be recorded. Results with a P-value less than 0.05 will be considered statistically significant.

Heterogeneity will be assessed using the chi-square-based Q test, and the I2 test will quantify the potential impact of heterogeneity on the meta-analysis. For chi-square values with P-value < 0.1, heterogeneity across the included studies will be considered statistically significant. An I² value of 0% to 40% represents “not important”; 30% to 60% represents “moderate heterogeneity”; 50% to 90% represents “substantial heterogeneity” (25). If the I^2^>50% and P ≤ 0.1, the random effects model will be used for meta-analysis. Any high heterogeneity will be explored through subgroup analysis or sensitivity analysis. Additionally, the study design and characteristics in the included studies will be analyzed.

### *A priori* subgroup analyses

If multiple studies reported homogenous outcomes in the following subgroups, planned subgroup analysis of the primary outcomes include the following:

1. Administration of neoadjuvant Tyrosine Kinase Inhibitors (TKIs) Prior to surgery: We will compare cases where TKIs were utilized before surgery to those where they were not. The application of neoadjuvant TKIs can effectively decrease tumor volume, subsequently downstage the clinical condition, and therefore limit the need for extensive surgical intervention, thus preventing unnecessary organ resection.
2. Use of adjuvant TKIs post-operation: The analysis will contrast outcomes from instances where TKIs were employed following the surgical procedure to those where they were not. The application of adjuvant therapy has demonstrated a substantial improvement in recurrence-free survival duration, indicating its suitability for patients with an intermediate to high risk of recurrence.
3. Propensity score analyses in non-randomized studies (NRS): The comparison will be between scenarios where propensity score analyses were undertaken versus where they were not. The implementation of propensity score analyses potentially leads to a relative balance in baseline characteristics, mitigating inherent biases.

### Sensitivity analysis

To exclude the situation that the results of the meta-analysis are substantially influenced by the presence of any individual study, we will conduct a sensitivity analysis by removing studies with a high risk of bias.

### Meta-biases and quality of evidence

When over ten studies are available, funnel plot will be used to examine publication bias (25). We will use the GRADE approach to assess the quality of findings systematically, which is considered an effective method to provide detailed information on assessments (26, 27). The quality of findings will be classified as high, moderate, low, and very low according to four dimensions: risk of bias, inconsistency, indirectness, and imprecision. High-quality findings will indicate a high grade of confidence in efficacy and quality of intervention. The GRADE assessments will be presented in a summary table.

## Discussion

Over the past decade, numerous studies have been published on the topic of local excision versus radical excision for rectal GISTs. However, the optimal choice between these surgical approaches remains a subject of debate. This review and meta-analysis aims to provide a comprehensive evaluation of the current evidence concerning the application of local excision and radical excision in patients with rectal GISTs. The findings from this review will serve as a foundation for future research and emphasize the implications for clinical practice.

## Data availability statement

The original contributions presented in the study are included in the article/**Supplementary Material**. Further inquiries can be directed to the corresponding authors.

## Author contributions

WL, CL, and LH provided equal contributions and shared co-first authorship. The original idea of this research was conceived by BZ, ZC, and LH. HZ, WL, and CS designed the protocol and drafted the manuscript. XY, MM, ZZ, CS, and TJ participated in developing the eligibility criteria, search strategy, and data extraction methods. WL registered the protocol in the International Prospective Register for Systematic Reviews and Meta-analysis. BZ and LH reviewed the manuscript. All authors contributed to the article and approved the submitted version.
