# Supplementary material for: Local excision and radical excision for rectal gastrointestinal stromal tumors: a meta-analysis protocol
Source: Front Oncol. 2023 Sep 7;13:1224725. doi: 10.3389/fonc.2023.1224725 (PMC10514207; doi:10.3389/fonc.2023.1224725)
Supplement: Supplementary file 1 [file DataSheet_1.docx]

**Search strategy in PubMed:** ("administration, rectal"[MeSH Terms] OR ("administration"[All Fields] AND "rectal"[All Fields]) OR "rectal administration"[All Fields] OR "rectal"[All Fields]) AND ("gastrointestinal stromal tumors"[MeSH Terms] OR ("gastrointestinal"[All Fields] AND "stromal"[All Fields] AND "tumors"[All Fields]) OR "gastrointestinal stromal tumors"[All Fields]) AND (("focal"[All Fields] OR "focalities"[All Fields] OR "focality"[All Fields] OR "focalization"[All Fields] OR "focalized"[All Fields] OR "focally"[All Fields] OR "focals"[All Fields] OR "local"[All Fields] OR "localisation"[All Fields] OR "localisations"[All Fields] OR "localise"[All Fields] OR "localised"[All Fields] OR "localises"[All Fields] OR "localising"[All Fields] OR "localization"[All Fields] OR "localizations"[All Fields] OR "localize"[All Fields] OR "localized"[All Fields] OR "localizer"[All Fields] OR "localizers"[All Fields] OR "localizes"[All Fields] OR "localizing"[All Fields] OR "locally"[All Fields] OR "locals"[All Fields]) AND ("excisable"[All Fields] OR "excise"[All Fields] OR "excised"[All Fields] OR "excises"[All Fields] OR "excising"[All Fields] OR "excision"[All Fields] OR "excisions"[All Fields]))
